# Shedding Light on the Dentition and Venom Delivery System of the Rear-Fanged Snake, *Galvarinus chilensis chilensis* (Serpentes: Dipsadidae: Tachymenini) from Chile

**DOI:** 10.3390/biology11121788

**Published:** 2022-12-08

**Authors:** Yarela Herrera, Sebastián Fuentes-Retamal, Ulrike Kemmerling, María Elisa Peichoto, Juan Carlos Ortiz, Félix A. Urra

**Affiliations:** 1 Metabolic Plasticity and Bioenergetics Laboratory, Program of Clinical and Molecular Pharmacology, Institute of Biomedical Sciences (ICBM), Faculty of Medicine, University of Chile, Santiago 8380453, Chile; 2 Network for Snake Venom Research and Drug Discovery, Santiago 8380453, Chile; 3 Program of Anatomy and Developmental Biology, Institute of Biomedical Sciences (ICBM), Faculty of Medicine, University of Chile, Santiago 8380453, Chile; 4 National Scientific and Technical Research Council (CONICET), National Institute of Tropical Medicine—National Administration of Laboratories and Health Institutes (ANLIS “Dr. Carlos G Malbrán”), Puerto Iguazú 9C59+8V, Misiones, Argentina; 5 Department of Zoology, Faculty of Natural and Oceanographic Sciences, University of Concepcion, Concepción 4070032, Chile

**Keywords:** rear-fanged snakes, colubrid, Duvernoy’s gland, opistoglyph, *Tachymenis*, ontogenetic variations

## Abstract

**Simple Summary:**

Studies about dentition and venom delivery systems (fang and gland) of rear-fanged snakes are limited. In Chile, the *Galvarinus* genus is represented by *G. chilensis* (formerly named *Tachymenis chilensis*), whose bites produce mild-to-moderate human envenomation. In this study, we describe the dentition and characteristics of fangs and their ontogenetic variations in *G. chilensis chilensis*. Moreover, histological and histochemistry analyses of the venom glands of this species are presented. Using micro-computed tomography and scanning electron microscopy, the dentitions of neonates, juveniles, and adults were analyzed. No ontogenetic and sex variations were observed. On the other hand, the fangs exhibited a groove with prominent ridges formed. Notably, the fang and groove lengths were significantly distinct between the three ontogenetic categories. However, we observed no differences between females and males. Using histological techniques, we found that the venom gland is close to the fangs, and the venom gland is constituted by acid mucous and serous acini, suggesting that it is a seromucous gland. Our results describe, for the first time, the distributional pattern and characteristics of the dentition and venom delivery system of the poorly studied snake *G. ch. chilensis.*

**Abstract:**

Although the rear-fanged snake *Galvarinus chilensis chilensis* (formerly named *Tachymenis ch. chilensis*) causes ophidian accidents with clinical importance in Chile, the anatomical and histological characterizations of the venom delivery system (venom gland and fang) of this species still remain unknown. This study describes the dentition and characteristics of fangs and their ontogenetic variations in *G. ch. chilensis*. Moreover, histological and histochemistry analyses of the venom glands of this species are presented. Using micro-computed tomography and scanning electron microscopy, the dentitions of neonates, juveniles, and adults were analyzed, and no ontogenetic variations in teeth length and number present in the dentary and maxilla were observed. Moreover, we found three types of basic teeth, with distributional patterns conserved in all ontogenetic categories. The fangs exhibited a groove from the base to the middle. At the end of the groove, prominent ridges are formed. The fang and groove lengths were significantly distinct between ontogenetic categories. No differences between females and males were observed. Histologically, we found that the venom gland is close to the fangs and has a seromucous composition. Our results describe, for the first time, the distributional pattern and characteristics of the dentition and venom delivery system of the poorly studied snake *G. ch. chilensis*.

## 1. Introduction

The morphological features of snake dentition are highly varied according to ecology and the evolutionary position in Serpentes lineages [1]. In the skull, four principal bones located in the upper (maxilla, palatine, and pterygoid) and lower (dentary) jaw have teeth [2]. However, in some basal lineages (booids and anilioids), there can also be premaxillary teeth (e.g., [3]). These are classified into four categories: basic, furrowed, grooved, or hollow tooth. Further, teeth exhibit additional specializations, such as multiple grooves, basal reinforcing ridges, development of a blade-like design, or secondary grooves [4]. The morphology of the basic teeth varies between recurved, curved, and linear shapes, whose role during the prey subjugation may be immobilization and retention, whereas other strategies (e.g., constriction or/and envenoming) produce the death of the prey [5].

A fang is an elongated maxilla tooth modified for inoculating venom to prey, and differs from other teeth by the presence of ridges along apical rostral and caudal surfaces [6]. Although fangs were previously considered a taxonomic criterion in snakes [2,7], it is now recognized that fang morphology is influenced by ecology and shares strikingly similar morphogenesis among front- and rear-fanged snakes, likely homologous [8]. It exhibits a high variability, being in some species a solid tooth, lacking a groove on the surface, thus no specialized tooth in inoculating venom (aglyphous teeth); a tooth located in the posterior bone maxilla (opisthoglyphous teeth); or a tooth located in the anterior maxilla position. In the latter case, they are tubular fangs with internal canals (proteroglyphous teeth) or are fangs significantly enlarged, subjected by the maxilla, independent of each other, with a duct that directs venom from the gland to the exterior (solenoglyphous teeth). Notably, there is abundant information on the fangs of venomous snake species with a high capacity for producing human fatalities, which contrasts with fang characterizations in opisthoglyphous species. Although the snakebites of these species produce very low human lethality in South America, it has been demonstrated that the venom contains several toxins evolutionarily related to those present in front-fanged snakes (e.g., [9,10,11,12,13,14]).

**Figure 1:**
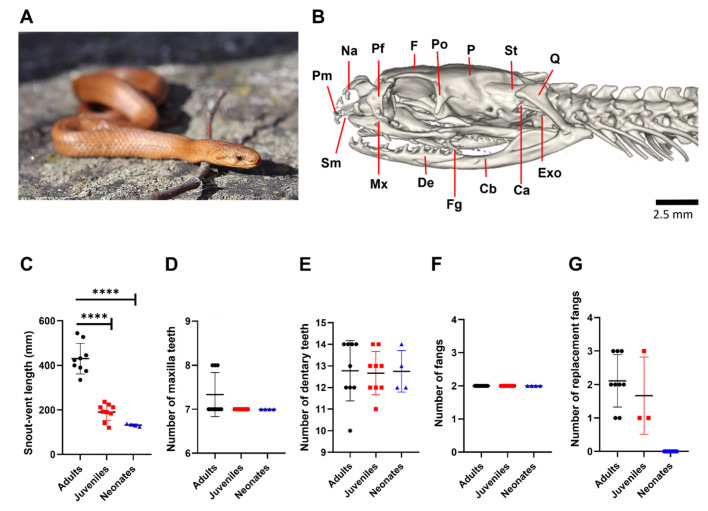
General features of the *Galvarinus ch. chilensis* dentition in different ontogenetic categories. (**A**) Image of an adult specimen from *Galvarinus ch. chilensis***.** (**B**) Cranial osteology in the left lateral (adult specimen, MZUC 45,730) from microCT image. (**C**) Snout–vent length; number of teeth in (**D**) maxilla and (**E**) dentary bones; (**F**) number of functional and (**G**) replacement fangs of different ontogenetic categories. Data shown are mean ± SD from adults (N = 10), juveniles (N = 9), and neonates (N = 4). Abbreviations of bones: Ca = columella auris; Cb = compound bone; De = dentary; Exo = exoccipital; F = frontal; Fg = fang; Mx = maxilla; Na = nasal; P = parietal; Pf = prefrontal; Pm = premaxilla; Po = postorbital; Q = quadrate; Sm = septomaxilla; St = supratemporal. **** *p* < 0.0001.

In Chile, the apparently low incidence of snakebites and lack of human mortality associated with snake envenoming [15,16] have led to the underestimation of morbidity and non-inclusion in official health programs [17,18]. An evident knowledge gap in toxicological and molecular aspects highlights the need for studying the characteristics of the venom delivery system of these rear-fanged snakes. Six opisthoglyph species belonging to *Incaspis*, *Philodryas*, *Pseudalsophis*, *Tachymenis,* and *Galvarinus* genera are recognized in Continental Chile [19,20], producing the bites of *G. chilensis chilensis* (formerly named *Tachymenis ch. chilensis*), with mild-to-moderate human envenomations [16]. Of them, *G. ch. chilensis* (Figure 1A) is the subspecies with the greatest distribution in Chilean territory [21,22], frequently sighted near places of human activities and responsible for several ophidian accidents. Despite the above, the venom properties [23] and histological characteristics of the venom gland [24] have been only studied in *T. peruviana* and for *G. ch. Chilensis*, and these characterizations remain unexplored.

Using micro-computed tomography, scanning electron microscopy, and histological analysis, we report the dentition and characteristics of fangs and their ontogenetic variations in *G. ch. chilensis*, contributing to the understanding of mechanisms involved in the human envenoming by this species.

## 2. Materials and Methods

### 2.1. Collection of Specimens

Three individuals of *G. ch. chilensis* (two adults and a juvenile) were collected in Ñuble’s Region, Comuna de Pinto, Chile. They were transported and kept in The Systematic and Conservation of Herpetozoos Laboratory, Universidad de Concepción, following the protocol described [25]. The collection and sampling of snakes were approved by Servicio Agricola y Ganadero (SAG) (Resolution No. 7466) and the bioethics and biosafety committee of Facultad de Ciencias Naturales y Oceanográficas, Universidad de Concepción (CEBB 1317-2022).

### 2.2. Scanning Electron Microscopy (SEM)

Twenty-three specimens of *G. ch. chilensis* from the Herpetology Collection from Museo de Zoología (Universidad de Concepción, Concepción, Chile) were used, which are detailed in Supplementary Table S1. The teeth anatomy was studied through scanning electron microscopy (SEM) JSM-6380 from Universidad de Concepción, according to Sánchez et al. [11]. The determination of dentary features in *G. ch. chilensis* was based on the literature [4,5,26], and measurements of length were made according to Britt et al. [27]. We determined the ontogenetic status according to Ortiz [28], Greene and Jaksic [29], and Urra et al. [21]. The snout–vent length for neonates: <138 mm (N = 4), juveniles: 138–236 mm (N = 9), and adults: >236 mm (N = 10). ImageJ software was used for the analysis of teeth characteristics and distance measurements. The anatomical nomenclature was based according to the literature [30,31].

### 2.3. Micro-Computed Tomography (Microct) Analysis

Five adult specimens of *G. ch. chilensis* were used (Supplementary Table S2). They were scanned through micro-CT. The computed tomography from skulls and teeth dentary was reconstructed using SkyScan1278, Bruker (Plataforma Experimental Bio-CT, Facultad de Odontología, Universidad de Chile), with a spatial resolution of 50µm, the smallest pixel size, and V.O.I studio (Multiple volumes of interest) in Dataviewer. Then, the pictures were obtained with CTvox. ImageJ software ver. 15.3 was used for teeth measurements.

### 2.4. Venom Gland Histology and Histochemistry

Venom glands were removed from two adult individuals of *G. ch. chilensis* (Supplementary Table S3) and fixed in 4% formaldehyde 0.1 M phosphate buffer (pH 7.3) for 24 h, dehydrated in alcohol, clarified in xylene, and embedded in paraffin. Section 5 µm in size were obtained and stained with hematoxylin-eosin, Alcian Blue 3% acetic acid (pH = 2.5), Alcian Blue 1% chlorohydric acid (pH = 1.0), periodic Acid-Schiff (PAS), and PAS-Alcian Blue 3% according to Suvarna et al. [32]. Samples were analyzed using an OLYMPUS BX43 microscope (Tokyo, Japan).

### 2.5. Statistical Analysis

All statistical analyses were performed using Graph Pad Prism 8.0.2 (GraphPad Software, San Diego, CA, USA). The data are expressed as the mean ± SD of at least four specimens. Statistical analysis was performed using one-way or two-way ANOVA with Bonferroni’s post-test for pairwise comparisons. The data were considered statistically significant at *p* < 0.05.

## 3. Results

### 3.1. G. ch. chilensis Has No Ontogenetic Differences in the Number of Teeth

Figure 1B shows the biomechanically important bones of the skull of *G. ch. chilensis,* maxilla (Mx), and dentary (De), accompanied by bones involved in jaw opening, such as supratemporal (St) and quadrate (Q). In the posterior area of the maxilla bone are positioned two fangs (Fg) that are backward-oriented with a pronounced curvature. We evaluated if the number of the maxilla and dentary tooth and venom-inoculating tooth exhibit variations according to three ontogenetic categories, neonates, juveniles, and adults, which were significantly different in the snout–vent length (SVL, Figure 1C). As Figure 1D–G shows, 7–8, 12–14, and 2 teeth were identified as the maxilla, dentary, and venom-inoculating teeth, respectively, in adult specimens. No differences between adults and juveniles or neonates were observed in these teeth. In contrast, the replacement fang was absent in newborn specimens (Figure 1G). Taken together, our results suggest that the number of maxillary and dentary teeth and fangs are invariable during the ontogenetic categories.

### 3.2. Tooth Types and Distances between the Dentary and Maxilla Have Unique Patterns and Are Independent of Ontogenetic Status in G. ch. chilensis

**Figure 2:**
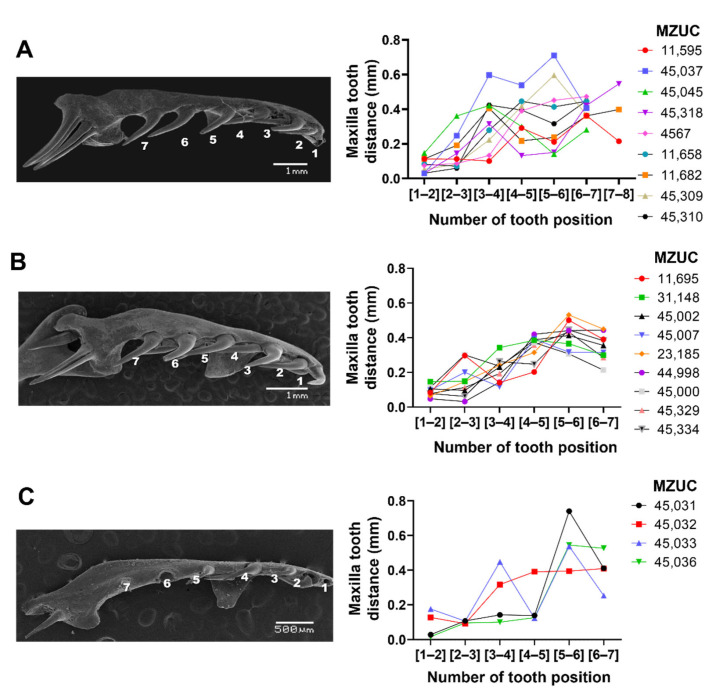
Variation of distances between teeth of the maxilla in (**A**) adults, (**B**) juveniles, and (**C**) neonates of *Galvarinus ch. chilensis* (labial view). The vouchers of specimens used for quantification are indicated in each graph. Data shown are mean ± SD from adults (N = 9), juveniles (N = 9), and neonates (N = 4). Representative images were obtained by scanning electron microscopy from (**A**) adult MZUC 45,037, (**B**) juvenile MZUC 11,695, and (**C**) neonate MZUC 45,032.

**Figure 3:**
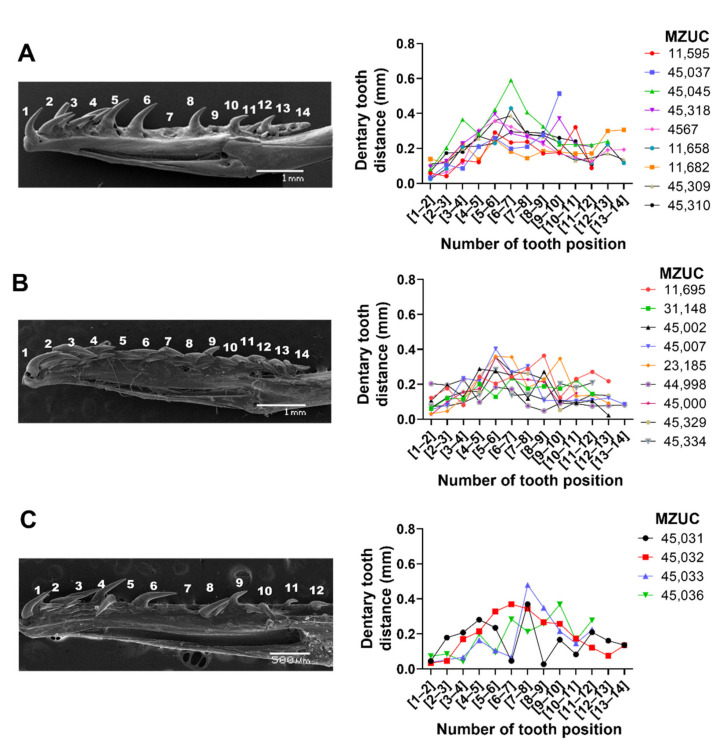
Variation of distances between teeth of the dentary in (**A**) adults, (**B**) juveniles, and (**C**) neonates of *Galvarinus ch. chilensis* (lingual view). The vouchers of specimens used for quantification are indicated in each graph. Data shown are mean ± SD from adults (N = 9), juveniles (N = 9), and neonates (N = 4). Representative images were obtained from (**A**) adult MZUC 11,658, (**B**) juvenile MZUC 45,007, and (**C**) neonate MZUC 45,033.

We evaluated whether the distances between the teeth in the dentary and maxilla vary according to the ontogenetic status of the individual. Using scanning electron microscopy, the distance between each maxillary and dentary tooth was quantified in *Galvarinus ch. chilensis* for the three ontogenetic categories and individual variations (Figure 2 and Figure 3). In the first instance, the distances between teeth for each specimen were studied and plotted individually according to their age group. In the maxilla (Figure 2), the posterior teeth have greater distances from each other than the anterior teeth. In the dentary bone (Figure 3), an increase in the distance of the teeth in the medial position (between tooth position 5 and 7), and a decrease in the distance in posterior positions were observed. No differences between males and females (in adults and juveniles) were observed (Supplementary Figure S2).

**Figure 4:**
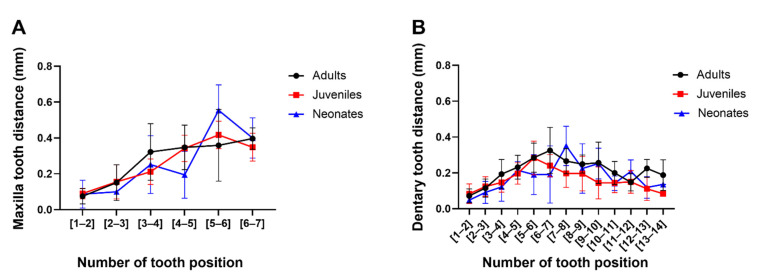
Variation of the distance between teeth of (**A**) maxilla and (**B**) dentary bones in *Galvarinus ch. chilensis*. Data shown are mean ± SD from adults (N = 9), juveniles (N = 9), and neonates (N = 4).

To determine ontogenetic differences, the individual values for adults, juveniles, and neonates were averaged. Notably, the distance between the maxillary teeth presents a different pattern than those between the dentary teeth. As Figure 4 shows, these tooth distance patterns were independent of ontogenetic status.

**Figure 5:**
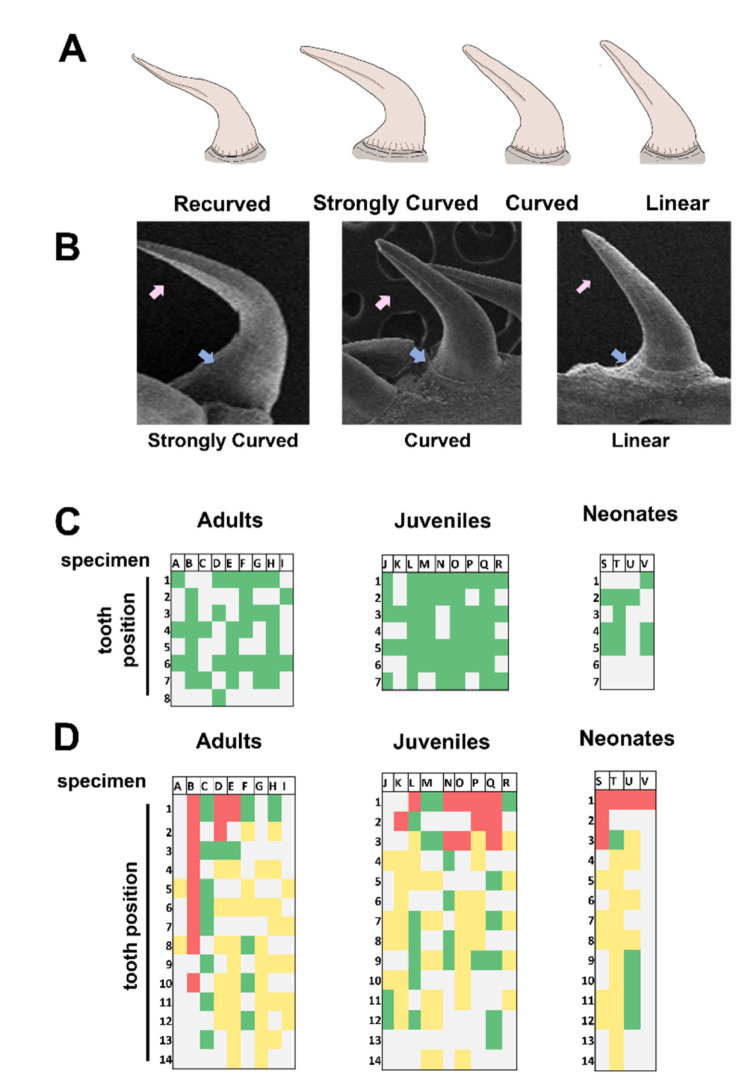
*Galvarinus ch. chilensis* presents three types of teeth the dentary and maxilla. (**A**) Representative illustration of tooth types in maxilla and dentary bones usually observed in snakes. (**B**) Representative images of strongly curved (adult—MZUC 11,658), curved (adult—MZUC 45,318), and linear (adult—MZUC 4567) teeth. (**C**) Distribution map of type of teeth in the maxilla and (**D**) dentary bones in adults (N = 9), juveniles (N = 9), and neonates (N = 4). Letters in boxes (A–V) indicate the MZUC specimens used. Detailed information and voucher numbers are shown in Supplementary Table S1. Colors represent the types of teeth as follows: green: strongly curved tooth; yellow: linear tooth; red: curved tooth; gray: not determined. Arrows indicate: pink = ridges and blue = parallel ridges.

**Figure 6:**
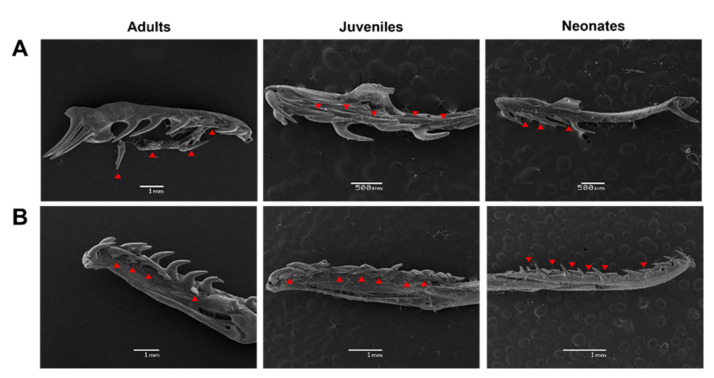
Presence of replacement tooth in maxilla and dentary bones. Red arrows indicate linear replacement teeth from (**A**) maxilla and (**B**) dentary bones. For (**A**), MZUC 45,037 (labial view), 45,000 (lingual view), and 45,036 (lingual view) were used; and for (**B**), MZUC 45,037 (lingual view), 44,998 (lingual view), and 45,032 (labial view) were used.

Figure 5A illustrates the tooth types usually observed in snakes [5]. We identified three basic tooth types in *G. ch. chilensis* (linear, curved, and strongly curved), and recurved teeth were absent in the three ontogenetic categories (Figure 5B,C). Strongly curved teeth predominate over others in the maxilla and anterior zone dentary bone; in contrast, linear teeth prevail in the dentary (in middle and posterior positions). Some specimens have curved teeth in dentary bones (Figure 5D). This represents a unique distributional pattern in maxilla and dentary bones, which is independent of ontogenetic categories. On the other hand, we observed several replacement teeth with linear characteristics in the lingual view (Figure 6).

### 3.3. Description of Fang Morphology of G. ch. chilensis

**Figure 7:**
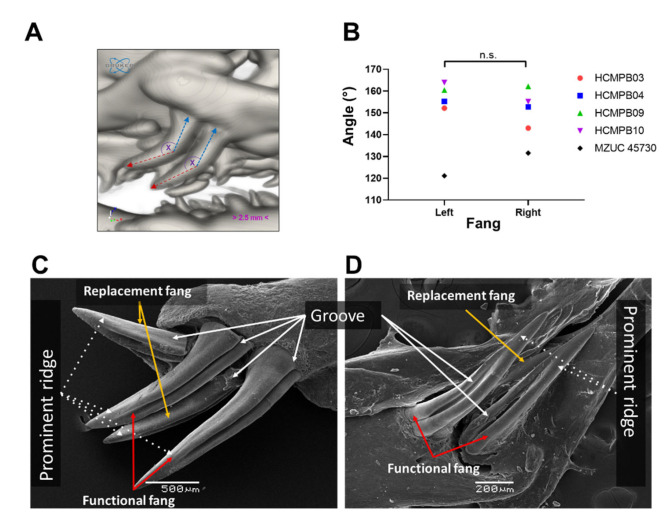
Anatomical and structural characteristics of the fang of *Galvarinus ch. chilensis*. (**A**,**B**) MicroCT image detailing the anatomical orientation and angle of fangs (adult, MZUC 45,730). SEM images show grooves and prominent ridges in (**C**) adult (MZUC 11,658) and (**D**) juvenile (MZUC 45,310) specimens. Abbreviation: n.s.: not significant.

**Figure 8:**
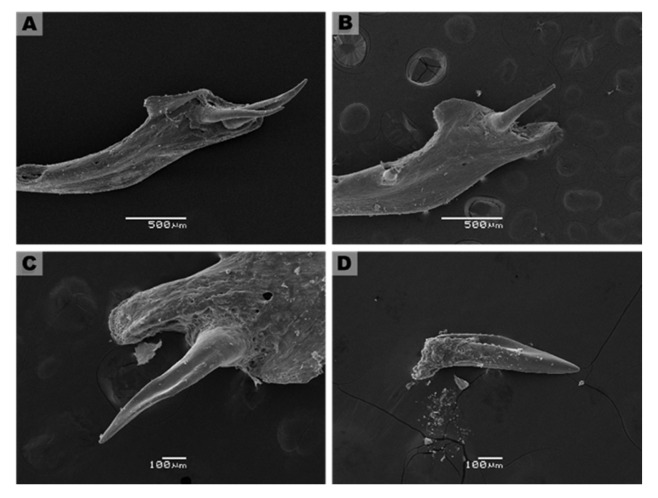
Anatomical and structural characteristics of the fang of neonate specimens of *Galvarinus ch. chilensis* (lingual view). Specimens used: MZUC (**A**) 45,031 (**B**) 45,032 (**C**) 45,033, and (**D**) 45,036.

Using microCT, we evaluated the orientation of fangs in intact skulls of adult specimens. The fangs are in the posterior zone of the maxilla bone and exhibit a 150° inclination towards the posterior area of the oral cavity, which does not have significant differences between the left and right fangs (left tooth: 150.6 ± 17.1° vs. right tooth: 148.9 ± 11.8°, N = 5, Figure 7A,B, and Supplementary Figure S1). Although each specimen had identical inclination angles for both teeth, two specimens showed slight differences (MZUC45730, HCMPB03). Figure 7C,D shows the generalities of the fangs for adult and juvenile specimens from SEM images. These are two elongated and functional fangs, accompanied by two smaller teeth with similar characteristics to functional fangs, suggesting that they are replacement fangs. In the frontal zone, the fang presents a groove along length teeth, from base to middle or 3/5 from total length towards the apex. At the end of the groove, prominent ridges are formed. These characteristics were observed in all adult and juvenile specimens studied (Figure 7C,D). The groove length is significantly distinct between the three ontogenetic categories. It is a shallow and incomplete groove in neonates and adults, a deep groove present throughout the tooth length. In neonates, individuals with fangs lacking grooves were observed (MZUC 45,031, 45,032, and 45,036; Figure 8).

**Figure 9:**
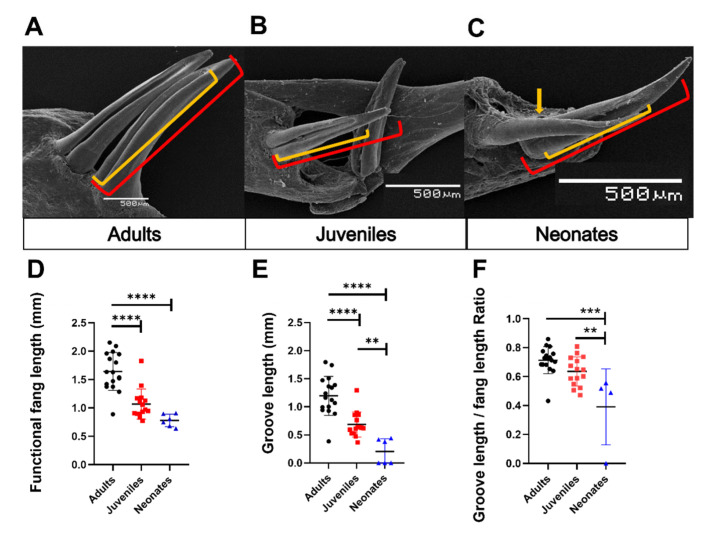
Ontogenetic variations of fang characteristics in *Galvarinus ch. chilensis*. (**A**–**C**) SEM images detailing the anatomical characteristics of fangs in adult (MZUC 45,037), juvenile (MZUC 45,334), and neonate (MZUC 45,031) specimens. Changes in the (**D**) functional fang length, (**E**) groove length, and (**F**) groove length/functional fang length ratio. Data shown are mean ± SD from all functional fangs (left and right) of adults (N = 10), juveniles (N = 9), and neonates (N = 4). ** *p* < 0.01, *** *p* < 0.001, **** *p* < 0.0001. Brackets indicate orange = groove and red = total fang length. The orange arrow indicates the absence of a groove.

We evaluated if the morphological characteristics of the teeth may be determined by ontogenetic status in *G. ch. chilensis* specimens, evaluating the fang length, groove length, and the groove length/fang length ratio as detailed in Figure 9A,C. As Figure 9D shows, the length of functional fang in *G. ch. chilensis* was significantly different in adults compared to juveniles (*p* < 0.0001) or neonates (*p* < 0.0001). However, there are no significant differences between juveniles and neonates (*p* = 0.1052). The groove lengths were significantly different in all the ontogenetic categories (Figure 9E). The functional/groove teeth ratio was differentially significant between adults and neonates (Figure 9F, *p* < 0.05). We did not observe differences between males and females in these parameters (Supplementary Figure S3).

### 3.4. Histological Characterization of Duvernoy’s Gland of G. ch. chilensis

**Figure 10:**
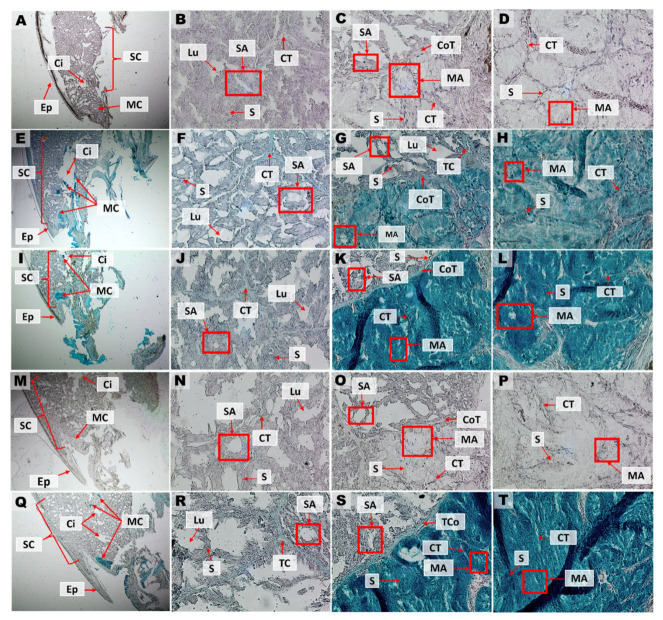
Histology and histochemistry of transverse cross-section from *G. ch. chilensis* venom gland. (**A**–**D**) Hematoxylin and eosin, (**E**–**H**) Alcian Blue 3% acetic acid, (**I**–**L**), Alcian Blue 1% chlorohydric acid, (**M**–**P**) PAS, (**Q**–**T**) PAS–Alcian Blue. Magnification: 4× for (**A**,**E**,**I**,**M**,**Q**) and 40× for the rest of the images. Abbreviations: Ci = cistern; CoT = connective tissue; CT = collector tube; Ep = epithelium; Lu = lumen; MA = mucous acinus; MC = mucous cells; S = septum; SA = serous acinus: SC = serous cells. All images are from an adult specimen (female, HCMPB07).

Macroscopically, the venom glands are localized in the lateral-posterior zone close to the fangs. Histological analysis showed that venom glands are delimited by a capsule of connective tissue and organized in lobules. Several small cisterns are distributed throughout the venom gland, which connect to a larger collector cistern located in the medial area of the gland (Figure 10A–D). These cells are negative for Alcian blue and PAS stainings, revealing their serous nature. The anterior and posterior zones of the venom gland reacted positively to Alcian Blue 3% (Figure 10E–H) and 1% (Figure 10I–L) stainings, suggesting the presence of mucopolysaccharides in structures specialized in the secretion of mucus. In the medial zone, the presence of mucous cells between serous cells around the collector cistern is observed. This evidence suggests a mixed composition in the gland, with mucous cells close to serous cells. PAS staining reacts positively in the epidermis; however, no reaction was observed in gland cells (Figure 10M–P). PAS–Alcian Blue (Figure 10Q–T) allows study in conjunction with reactions observed with Alcian blue 3% and PAS together. Similar histological aspects were observed in another adult specimen (male) of *G. ch. chilensis* (Supplementary Figure S4).

## 4. Discussion

Dipsadidae is one the most diverse families of South American rear-fanged snakes [33], within which the Tachymenini tribe is widely distributed throughout South America, presenting very distinctive phenotypes, habitats, and behaviors. It comprises 36 species whose venom delivery systems have been scarcely studied [20]. A recent taxonomic study revealed a complex evolutionary relationship of the species belonging to this tribe, separating *Tachymenis* species into two different genera, *Tachymenis* (*T. ocellata*, *T. peruviana*, and *T. trigona)* and *Galvarinus* (*G. attenuatus*, *G. tarmensis*, and *G. chilensis*, with two subspecies: *G. ch. coronellina* and *G. ch. chilensis*), corresponding to the previously known “*peruviana*” and “*chilensis*” groups, respectively [20].

In Chile, *Galvarinus ch. chilensis* is one of the main snake species responsible for causing ophidian accidents [16]; however, several aspects of venom composition and fang characterization remain poorly understood. This species reproduces viviparously [21], has cathemeral habits [34,35], and preys on anurans and lizards [29]. Notably, *G. ch. chilensis* exhibits a known defensive behavior to human approaching and handling, exhibiting an open mouth and attempting to bite [36], which could lead to human envenomation [37,38]. This highlights the relevant role of dentition and the venom delivery system in the natural history of *G. ch. chilensis*.

It is recognized that tooth features can vary between snake species [39], having the dental ridges functions such as cutting the interior from prey and helping the hitch of teeth [5]. A correlation of dental morphology with dietary preferences has been extensively demonstrated [1,27,40,41], suggesting that specialists exhibit a greater number of dental characteristics [42]. Moreover, differences in dental morphology can be the result of sexual dimorphism and ecology in snakes [6,43,44]. In *Pseudaspis cana*, males have more significant maxillary tooth variation, enlargement of the posterior-most maxillary teeth, and dentary teeth with posterior carinae compared to females [6]. On the other hand, a convergence in fang shape in snake species with similar diets and more differences in the fang morphology in closely related species with different diets have been described [45]. Our results suggest that the tooth number is constant in the three ontogenetic categories studied and agree with a previous report [46]. In dentary and maxilla teeth, a ridge is accompanied by parallel ridges in the basic tooth of the neonate, juvenile, and adult specimens of *G. ch. chilensis*. In addition, we identified three basic tooth types in *G. ch. chilensis*, exhibiting a unique distributional pattern in maxilla and dentary bones, which was independent of ontogenetic categories. Notably, strongly curved teeth predominated in the maxilla. The teeth present in the dentary bone were strongly curved (in the first positions) and linear (in the middle and posterior positions). The strongly curved teeth in the anterior position may prevent the prey from escaping because if the prey attempts to move out of the mouth, the teeth will penetrate deeper and securely impale the prey item [1]. Each tooth in both bones exhibited different patterns of distance, which was independent of sex and ontogenetic categories. To our knowledge, this is the first characterization of the teeth in the dentary and maxilla in the different ontogenetic status of *G. ch. chilensis*.

In contrast to other Tachymenini tribe species, we found that adult specimens of *G. ch. chilensis* have two elongated and curved backward fangs with angles of 148.93° ± 11.88 (left tooth) to 150.58° ± 17.08 (right tooth), which are accompanied by two smaller and linear teeth with similar characteristics (replacement fangs). The most significant variation in the inclination angle of fang was observed in the adult specimen MZUC45,730 (locality: Chiloé Island, Chile), which corresponds to the southernmost distributed population of this species [22]. Further studies are needed to verify whether geographic distribution determines variations in the morphology and characteristics of fangs. Notably, our results suggest that the maturation process from replacement to functional fang involves changes in its curvature. On the other hand, ontogenetic differences were observed in the fang length and morphology, which were independent of sex. The early ontogenetic stage has a linear fang with a shallow groove (which sometimes is absent) and a high fang length/groove length ratio, and in the adult specimens, we found a deeper and more extensive groove. These changes also involved a progressive curvature in the fang. Usually, ontogenetic changes in tooth morphology are associated with a dietary shift in snakes. For example, juveniles have robust and blunter fangs, probably feeding on scaly lizards, whereas adult specimens display narrow fangs with sharper tips to feed on softer mammalian prey [45,47]. Since *G. ch. chilensis* lacks an ontogenetic shift in the diet [29], we are unable to associate the changes in the fang morphology with any feature known in the natural history of this species. Future studies on the variation of venom composition during the ontogeny of *G. ch. chilensis* may explain these structural changes reported by us.

Currently, the venom composition of *G. ch chilensis* is unknown, but clinical manifestations of human bites suggest the presence of toxins with anti-coagulant, pro-inflammatory, and edematogenic effects [37,38,48]. Additionally, it has been demonstrated that this species feed on toxic toads such as *Rhinella rubropunctata* [49], which suggests that the venom of this snake species may help to rapidly subdue toxic prey (probably avoiding the release of their toxins). In this work, we describe the venom gland of *G. ch. chilensis* with a histological organization similar to that described in other rear-fanged snakes such as *Philodryas olfersii* [50], *Helicops modestus* [51], *Erythrolamprus aesculapii* [12], *Leptophis ahaetulla marginatus* [11], and *Leptodeira annulata* [52]. The venom gland of rear-fanged snakes can be classified into four histologically different types [53]: (1) the presence of mucous cells in the supralabial region without the venom gland; (2) predominant serous cells with mucous intercalate in the venom gland (mixed venom gland or seromucous); (3) predominant mucous with limited serous cells in gland; and (4) exclusively serous cells in the gland. Our histochemistry analysis indicates that *G. ch. chilensis* has a mixed venom gland, a characteristic reported in other Neotropical rear-fanged snakes [12,50,51,53,54,55,56]. This venom gland exhibited a seromucous composition, lacking the melanin pigmentation that has been described for some diurnal rear-fanged snakes [11,57].

Supporting the fact that both the fang and venom secretion of *G. ch chilensis* must play a key role in subduing its prey, we showed that the former is grooved (the later ontogenetic stage, the deepest groove) to facilitate the venom injection into prey. This is different from *Xenodon merremii*, which is another dipsadid species that also feed on toads from the genus *Rhinella* and is immune to their cutaneous toxins [58], but this has only enlarged teeth (without any groove) at the posterior end of the maxilla to aid prey manipulation and chewing to inject venom before swallowing [59]. This demonstrates that diet is not the only parameter determining the dentition and venom-delivery system of rear-fanged snakes.

## 5. Conclusions

*Galvarinus ch. chilensis* is a poorly known snake that causes ophidian accidents with clinical relevance in Chile. Despite this, the characteristics of its dentition and venom delivery system are unknown. In this work, we showed that maxillary teeth are predominantly strongly curved, and the teeth present in the dentary bone are strongly curved (in the first positions) and linear (in the middle and posterior positions). Additionally, each tooth in both bones exhibits different patterns of distance. All these characteristics were independent of sex and ontogenetic categories. On the other hand, the fangs are oriented towards the posterior area in the oral cavity with a curvature of 150 degrees. The fangs exhibited a deep groove with prominent ridges, which showed variations dependent on the ontogenetic categories. The fangs are connected to venom glands, which are delimited by connective tissue and organized in lobules, which are serous. Several small cisterns connected to a central collector tube showed mucous cells, suggesting a seromucous composition of the venom gland. This is the first description of the dentition and venom delivery system for *G. ch. chilensis*.

## Data Availability

The data is contained within the article or Supplementary Materials.

## Supplementary Materials

The following supporting information can be downloaded at: https://www.mdpi.com/article/10.3390/biology11121788/s1, Figure S1. View of left and right fangs of adult specimens of *G. ch. chilensis* using computerized microtomography. Figure S2. Variation of the distance between teeth in males and females. Figure S3. Variation of the characteristics of fang and groove in males and females. Figure S4. Histology and histochemistry of transverse cross-section of venom gland from another adult studied. Table S1. Specimens used in scanning electron microscopy. Table S2. Specimens used in microtomography computerized. Table S3. Specimens used for histochemistry.
