# Supplementary material for: Shedding Light on the Dentition and Venom Delivery System of the Rear-Fanged Snake, Galvarinus chilensis chilensis (Serpentes: Dipsadidae: Tachymenini) from Chile
Source: Biology (Basel). 2022 Dec 8;11(12):1788. doi: 10.3390/biology11121788 (PMC9775764; doi:10.3390/biology11121788)
Supplement: Supplementary file 1 [file biology-11-01788-s001.zip › biology-1965061-supplementary.pdf]

Shedding lights on the dentition and venom-delivery system of the rear-fanged snake, *Galvarinus chilensis chilensis* (Serpentes: Dipsadidae: Tachymenini) from Chile

Yarela Herrera, Sebastián Fuentes-Retamal, Ulrike Kemmerling, María Elisa Peichoto, Juan Carlos Ortiz & Félix A. Urra

Supplementary information

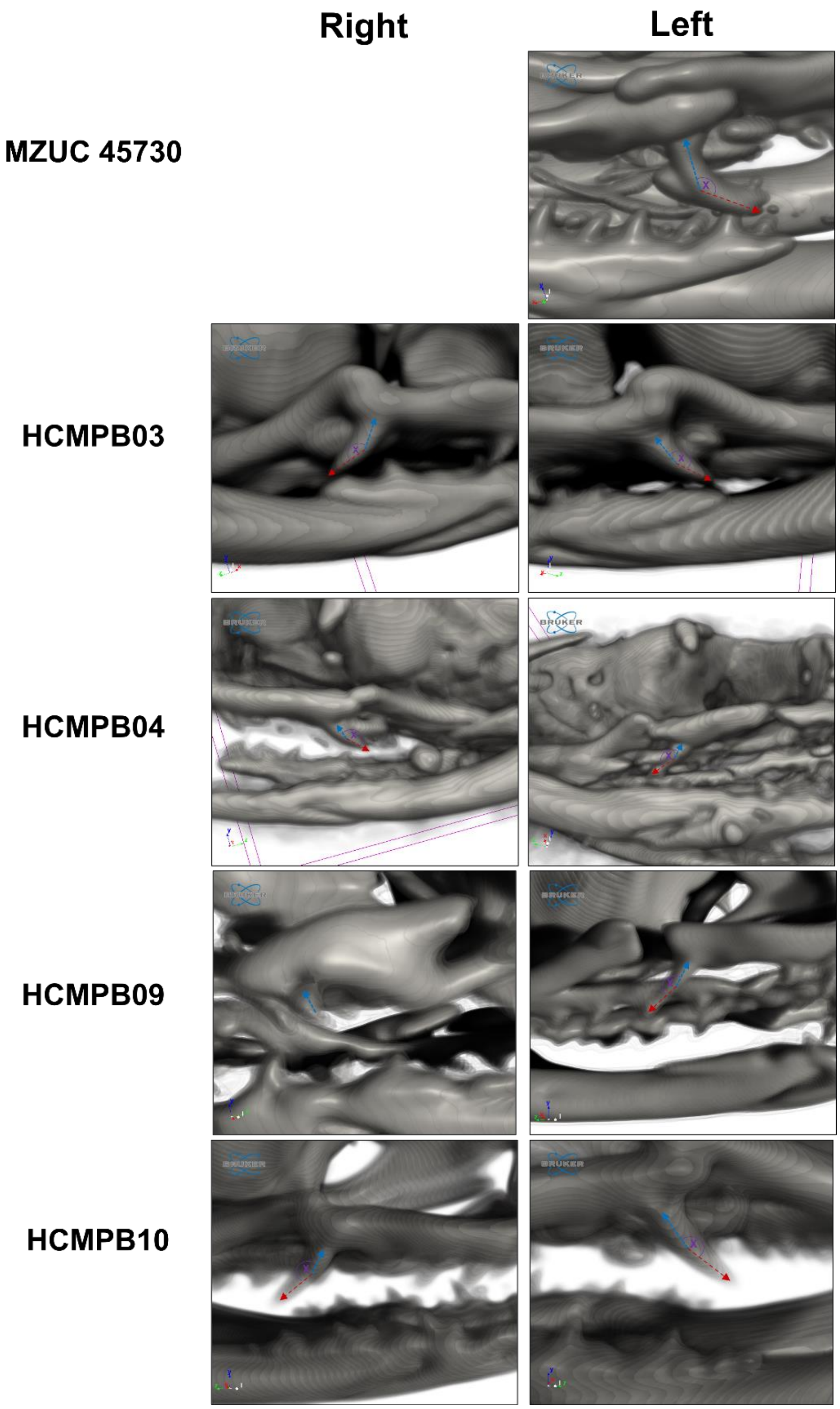

Supplementary Figure S1. View of left and right fangs of adult specimens of *G. ch. chilensis* using computerized microtomography. The right fang of MZUC 45730 specimen is shown in Figure 5.

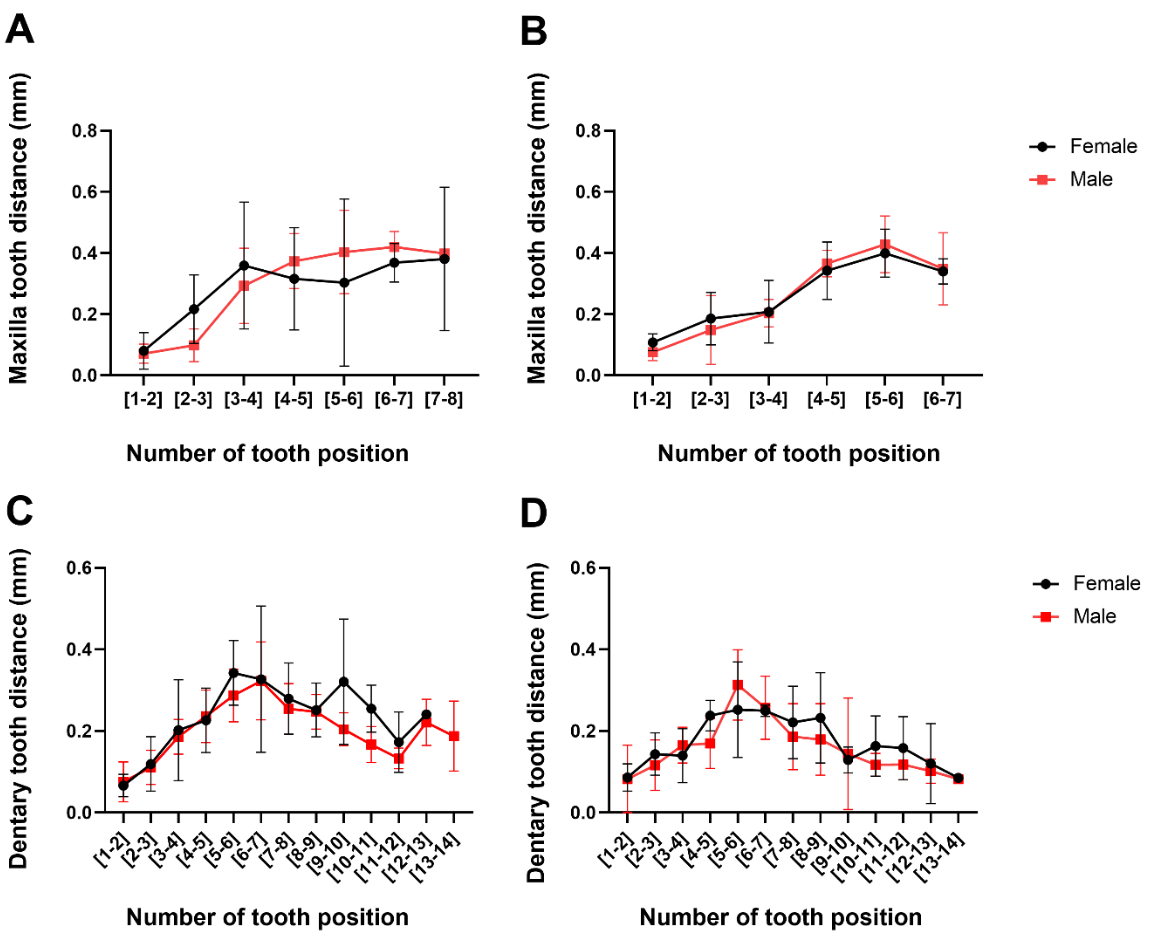

Supplementary Figure S2. Variation of the distance between teeth of (**A-B**) maxilla and (**C-D**) dentary bones in females and males of *Galvarinus ch. chilensis* in (**A, C**) adults and (**B, D**) juvenile specimens. Data shown are mean ± SD from adults (males: N=5, females: N=4) and juveniles (males: N=4, females: N=4).

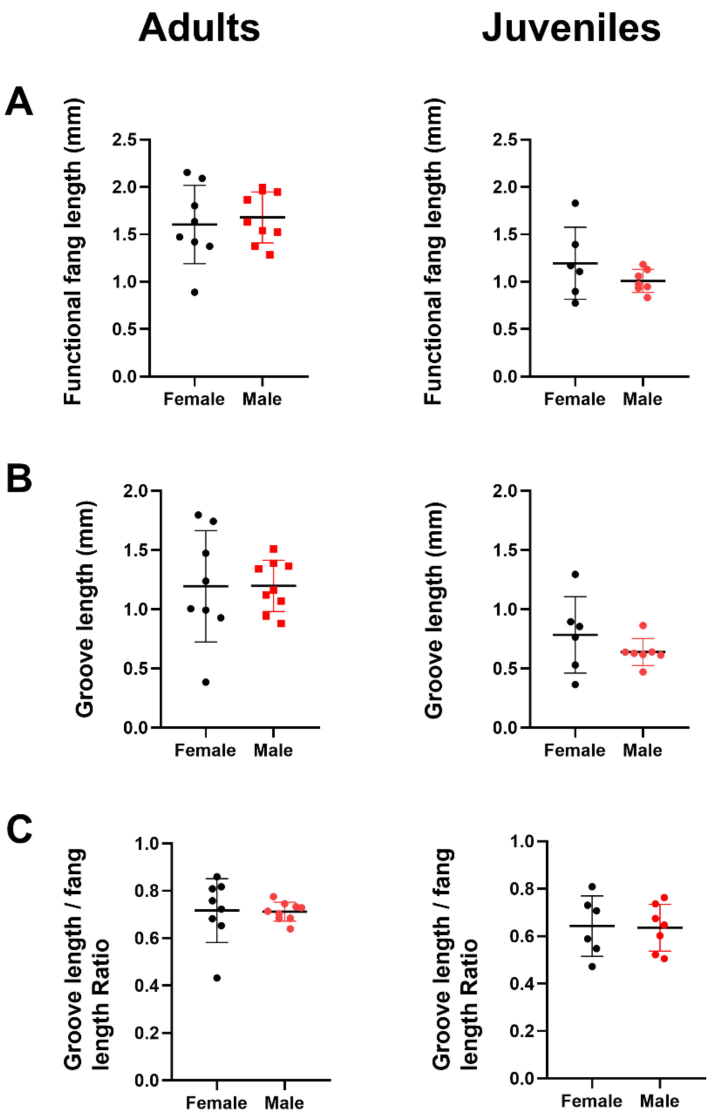

Supplementary Figure S3. Changes in the (**A**) Functional fang, (**B**) Groove length, and (**C**) Groove length / functional fang length ratio between females and males. Data shown are mean ± SD from all functional fangs (left and right) of adults (males: N=5, females: N=5) and juveniles (males: N=4, females: N=4).

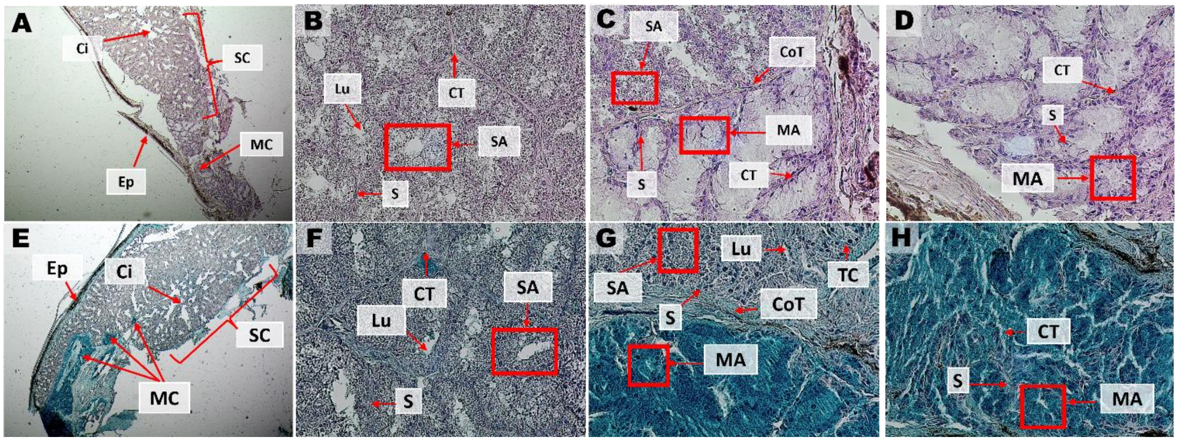

Supplementary Figure S4. Histology and histochemistry of a transverse cross-section from *G. ch. chilensis* venom gland. (**A - D**) Hematoxylin/Eosin and (**E - H**) Alcian Blue 3%, (I- L). Magnification 4X for **A**, **E** and 40X for the rest of the images, Abbreviations: Ci= Cistern; CoT= connective tissue; CT= collector tube; Ep = Epithelium; Lu= Lumen; MA= Mucous acinus; MC= Mucous cells; S= Septum; SA= Serous acinus: SC= Serous cells. All images are from an adult specimen (male, HCMPB05).

Supplementary Table S1. Specimens used in scanning electron microscopy. Abbreviations: MZUC= Museo de Zoología, Universidad de Concepción; O. C= Ontogenetic categories; Fig. 5 letters= Letters in boxes (A-V) of Figure 5 indicate the MZUC specimens used, detailed information and voucher numbers are shown in this table.

**Specie**	**Collection**	**Number**	**O. C**	**Sex**	**Fig, 5 letters**	**Location**
G. chilensis	MZUC	11595	Adult	Female	A	Chile - Biobío's Region - Provincia de Arauco - Laraquete - Las Cruces
G. chilensis	MZUC	45037	Adult	Female	B	Chile - Biobío's Region - Provincia de Arauco – Ramadilla
G. chilensis	MZUC	45045	Adult	Female	C	Chile - Biobío's Region - Provincia de Arauco - Ramadilla
G. chilensis	MZUC	45048	Adult	Female	-	Chile - Biobío's Region - Provincia de Arauco - Ramadilla
G. chilensis	MZUC	45318	Adult	Female	D	Chile - Biobío's Region - Los Ángeles
G. chilensis	MZUC	4567	Adult	Male	E	Chile - Biobío's Region - Provincia de Arauco - Ramadilla
G. chilensis	MZUC	11658	Adult	Male	F	Chile - Biobío's Region - Provincia de Arauco - Ramadilla
G. chilensis	MZUC	11682	Adult	Male	G	Chile - Biobío's Region - Provincia de Arauco - Ramadilla
G. chilensis	MZUC	45309	Adult	Male	H	Chile - Biobío's Region – Provincia de Concepción - Surroundings
G. chilensis	MZUC	45310	Adult	Male	I	Chile - Biobío's Region - Provincia de Concepción - Surroundings
G. chilensis	MZUC	11695	Juvenil	Female	J	Chile - Biobío's Region - Provincia de Concepción- Concepción
G. chilensis	MZUC	31148	Juvenil	Female	K	Chile - Biobío's Region – Parque Nacional Nahuelbuta
G. chilensis	MZUC	45002	Juvenil	Female	L	Chile - Biobío's Region – Provincia de Malleco - Antuco Volcano - Los Barros
G. chilensis	MZUC	45007	Juvenil	Female	M	Chile - Biobío's Region - Provincia de Malleco – Antuco Volcano - Los Barros
G. chilensis	MZUC	23185	Juvenil	Male	N	Chile - Biobío's Region - Dichato
G. chilensis	MZUC	44998	Juvenil	Male	O	Chile - Biobío's Region - Provincia de Malleco - Antuco Volcano - Los Barros
G. chilensis	MZUC	45000	Juvenil	Male	P	Chile - Biobío's Region - Provincia de Malleco - Antuco Volcano - Los Barros
G. chilensis	MZUC	45329	Juvenil	Male	Q	Chile - Biobío's Region - San Fabian de Alico – Río Ñuble
G. chilensis	MZUC	45334	Juvenil	Unidentified	R	Chile - Biobío's Region - Provincia de Arauco – Planta Horcones
G. chilensis	MZUC	45031	Neonate	Unidentified	S	Chile - Biobío's Region - Provincia de Arauco - Laraquete - Las Cruces
G. chilensis	MZUC	45032	Neonate	Unidentified	T	Chile - Biobío's Region – Provincia de Arauco - Laraquete - Las Cruces

G. chilensis	MZUC	45033	Neonate	Unidentified	U	Chile - Biobío's Region - Arauco Province - Laraquete - Las Cruces
G. chilensis	MZUC	45036	Neonate	Unidentified	V	Chile - Biobío's Region - Provincia Arauco - Laraquete - Las Cruces

Supplementary Table S2. Specimens used in microtomography computerized. Abbreviations: HCMPB= Herpetological collection – MPB Lab (U. Chile); MZUC= Museo de Zoología, Universidad de Concepción; O. C= Ontogenetic categories

**Specie**	**Collection**	**Number**	**O. C**	**Sex**	**Location**
G. chilensis	HCMPB	3	Adult	Unidentified	Chile – Ñuble's Region – Comuna de Pinto – Shangri-la
G. chilensis	HCMPB	4	Juvenile	Unidentified	Chile – Ñuble's Region – Comuna de Pinto – Shangri-la
G. chilensis	HCMPB	9	Adult	Female	Chile - Ñuble's Region - Comuna de Pinto
G. chilensis	HCMPB	10	Adult	Male	Chile - Ñuble's Region - Comuna de Pinto
G. chilensis	MZUC	45730	Adult	Male	Chile - Los Lagos's Region – Provincia de Chiloé

Supplementary Table S3. Specimens used for histochemistry. Abbreviation: HCMPB= Herpetological collection – MPB Lab (U. Chile); O. C= Ontogenetic categories

**Specie**	**Collection**	**Number**	**O. C**	**Sex**	**Location**
G. chilensis	HCMPB	5	Adult	Male	Chile - Región del Ñuble - Comuna de Pinto
G. chilensis	HCMPB	7	Adult	Female	Chile - Región del Ñuble - Comuna de Pinto
